# Clustering and Characterization of the Lactation Curves of Dairy Cows Using *K*-Medoids Clustering Algorithm

**DOI:** 10.3390/ani10081348

**Published:** 2020-08-04

**Authors:** Mingyung Lee, Seonghun Lee, Jaehwa Park, Seongwon Seo

**Affiliations:** 1 Division of Animal and Dairy Sciences, Chungnam National University, 99 Daehak-ro, Yuseong-gu, Daejeon 34134, Korea; mingyung1203@cnu.kr; 2 Department of Computer Science and Engineering, Chung-Ang University, 84 Heukseok-ro, Dongjak-gu, Seoul 06974, Korea; gnstjdok@cau.ac.kr

**Keywords:** dairy cow, lactation curve, *k*-medoids clustering, milking characteristics, model fitting

## Abstract

**Simple Summary:**

A lactation curve (LC) provides valuable insights in planning appropriate management strategies related to health, nutrition, and breeding in dairy cows. A clustering based approach on LC patterns analysis is presented. The *k*-medoids algorithm is adopted for the clustering. This approach generates several clusters which have similar milking characteristics of total milk yield, peak milk yield, and days in milk at peak yield. The LCs of some groups represent characteristics of atypical milking patterns which are not considered much in previous approaches, whereas LCs of the other groups show the typical LC patterns similar to the results of previous methods. This approach could be used as a tool to manage an abnormal herd of cows.

**Abstract:**

The aim of the study was to group the lactation curve (LC) of Holstein cows in several clusters based on their milking characteristics and to investigate physiological differences among the clusters. Milking data of 330 lactations which have a milk yield per day during entire lactation period were used. The data were obtained by refinement from 1332 lactations from 724 cows collected from commercial farms. Based on the similarity measures, clustering was performed using the *k*-medoids algorithm; the number of clusters was determined to be six, following the elbow method. Significant differences on parity, peak milk yield, DIM at peak milk yield, and average and total milk yield (*p* < 0.01) were observed among the clusters. Four clusters, which include 82% of data, show typical LC patterns. The other two clusters represent atypical patterns. Comparing to the LCs generated from the previous models, Wood, Wilmink and Dijsktra, it is observed that the prediction errors in the atypical patterns of the two clusters are much larger than those of the other four cases of typical patterns. The presented model can be used as a tool to refine characterization on the typical LC patterns, excluding atypical patterns as exceptional cases.

## 1. Introduction

Lactation curves (LC) provide invaluable information that could be used to evaluate the genetic and management status of lactating dairy cows. An LC is a graphical representation of change in milk yield during the lactation period following calving [1]. It typically increases rapidly until the peak yield is achieved and then decreases slowly until the drying period. The shape of an LC, described based on peak yield, peak time, and persistency, is used to determine the milking potential of a dairy cow and is an index that facilitates feed and health management, in addition to breeding decision [2,3]. For a large group of cows, the mean LC follows a typical pattern, which can be described mathematically using a simple equation [4]. Various equations have been proposed [5,6,7,8], and they fit the mean LC of a large group of cows relatively well [2,9].

The shapes of individual LC, however, are quite diverse because LC shape varies based on various factors, including biological factors such as breed, genetic effects, physiological conditions, pregnancy, parity, and age, and environmental factors such as feed, herd management, and calving season [5,10,11,12,13]. Some studies have reported atypical shapes, such as the absence of peak yield, in approximately 20∼30% of cases [9,14,15]. Despite their considerable proportion, such cases have been considered outliers or disregarded in analyses, as they are difficult to explain using previously developed equations [16,17,18]. To facilitate precision dairy farming, however, it is useful to identify and analyze LC shapes that can be influenced by individual variation, feed management, or disease. In previous studies, LCs have been analyzed after grouping dairy cows based on production levels, parity differences, or presence of disease [3,19,20,21]. But the limitations remain because those studies tried to derive LCs for predefined groups instead of extracting physiological characteristics from various LCs.

Clustering analysis facilitates the defining of groups objectively based on phenotypic observations. Particularly, the unsupervised and non-hierarchical clustering methods (i.e., *k*-means and *k*-medoids) are extensively used for the analysis of large datasets because they are powerful and have lower computational cost requirements than hierarchical clustering methods [22,23]. Several studies in the field of dairy science have applied the *k*-means method to classify dairy cows according to genetic status and health status based on peak milk yield, milk components, and blood properties [24,25,26,27].

The objective of the present paper, therefore, was to cluster lactation data based on the shapes of LCs using the *k*-medoids, a variant of *k*-means, clustering algorithm. We obtained three years of milking records from commercial Korean dairy farms and used them for the analyses. After clustering, the representative LCs of each cluster were fitted using several conventional LC models. We also investigated differences in physiological and milking characteristics among the clustered groups.

## 2. Backgrounds

An LC can be thought as a time-series of daily milk production for an individual dairy cattle. Therefore, grouping LCs can be handled by time-series clustering. Time-series clustering and its application have been widely studied in various fields [28,29,30,31,32]. Liao T. W. [28] provided guidance on how to apply clustering algorithms to time-series data. Aghabozorgi et al. [31] also classified the time-series clustering into three categories: whole time-series clustering, subsequence time-series clustering and time point clustering. In the study, the authors only focused to whole time-series clustering because subsequence time-series clustering was considered meaningless by Keogh and Lin [33] and time point clustering is similar to time-series segmentation [31].

There are two most extensively used time-series clustering algorithms: the connectivity-based hierarchical clustering and the centroid-based *k*-means clustering. Hierarchical clustering repeatedly merges to form a larger cluster (i.e., agglomerative clustering) or divides a larger cluster into smaller clusters until the cluster is not partitioned (i.e., divisive clustering). This clustering algorithm is often used as a primary prescription for time-series clustering, because it requires no prior understanding of the datasets and clusters [31]. However, due to its high computational complexity, the hierarchical clustering method is not recommended for large datasets [28,32]. Conversely, *k*-means algorithm is a non-trivial algorithm, which implies that it relies heavily on the initial center of the cluster [31,34]. In addition, *k*-means algorithm has a slow initial convergence rate [34]. To address the challenge, a weighted probability distribution can be adopted to initialize the center of the cluster, which is introduced in the *k*-means++ algorithm [34]. In the method, the initial center is selected with probability $P\left(x\right)=D{\left(x\right)}^{2}/\sum_{x^{\prime }\in X}D{\left(x^{\prime }\right)}^{2}$, where D(x) is the shortest distance from the data point to the nearest center already selected. Therefore, the method increases the rate of convergence, and evenly distributes the initial centers.

Although the *k*-means algorithm is the most extensively applied, the *k*-medoids algorithm has promising applications because it is more robust against noise and outliers [23,35]. For example, Sauder et al. [36] compared the performance of hierarchical clustering and two non-hierarchical clustering algorithms (i.e., *k*-means and *k*-medoids) in the classification of Holstein dairy cows based on their growth curves, and reported that the *k*-medoids method was the most appropriate for grouping cows. They also observed significant differences in milking performance between the clustered groups.

*K*-medoids algorithm is a *k*-means variant that complements the noise vulnerabilities of *k*-means algorithm. *K*-means algorithm adopts the mean value as the center of the cluster. Therefore, it naturally suffers from noise [32]. Conversely, *k*-medoids algorithm is less sensitive to outliers because it uses the median for the selection of center values, which makes the computation procedure in *k*-medoids more complex than the procedure in *k*-means algorithm; however, *k*-medoids is still a more powerful tool for clustering large datasets than hierarchical clustering [31]. In addition, unlike *k*-means clustering, *k*-medoids does not require additional calculation for the inter-LC distances whenever the center is updated.

## 3. Materials and Methods

### 3.1. Dataset

The datasets were collected from January 2016 to December 2018 from four commercial farms in Chungcheong Province, South Korea. An automatic milking system (*Astronaut A4*; Lely Industries NV, Maassluis, the Netherlands) was installed in three farms, while a conventional milking parlor system (DeLaval international AB, Tumba, Sweden) was used in one farm. Milk yield data were stored individually using radio-frequency identification (RFID) tags in both milking systems. A total of 1332 lactations (i.e., records of milk production events during a single lactation period) were recorded from 724 cows (Table 1).

**Figure 1:**
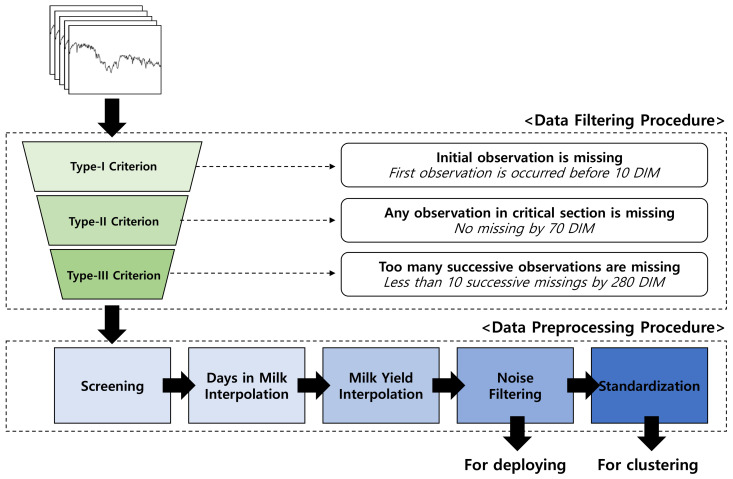
Data filtering and preprocessing procedure.

The milking records from 10 to 280 days-in-milk (DIM; calving day was considered as 0 DIM) were used. This was because the data before 10 DIM and after 280 DIM were error-prone due to differences in management practices among the four farms. In addition, an ideal LC is formed using a concave function and the peak is often achieved within 10 weeks [37]. Because the peak information is critical source of information in LCs, we set the non-interpolation period within the 10 through 70 DIM. Lactation records were preprocessed according to three criteria for incomplete data (Figure 1).

First of all, data recorded prior to 10 DIM were filtered out (Type-I). Datasets which had any missing records between 10 to 70 DIM were also discarded (Type-II). Finally, lactations that contained missing data for more than 10 consecutive days between 70 to 280 DIM were also discarded dataset (Type-III).

Out of the total 1332 lactation data, 228 (22.8%), 42 (4.2%), and 732 (73.1%) records were excluded via the Type-I, II and III criteria, respectively. Consequently, a total of 330 (24.74%) lactation records remained in the final dataset (Table 2). One-hundred and one (33.6%) records were from primiparous cows (i.e., cows at first lactation), with an average parity of 2.3, which is close to the Korean national average parity [38].

Data preprocessing was performed on the final dataset to fill gaps and minimize noise in the data. Missing values in lactation records were compensated for via the linear interpolation method [39]. Table 2 presents lactation performance values within the dataset. The milking days, average daily milk yield per head, and total milk yield in a lactation increased with an increase in parity number. After the compensation step, noise filtering was also performed. The moving average was used based on a 10-day window.

Before the clustering, the datasets were normalized using Z-score transformation. The normalization process minimizes clustering divergence caused by differences in dynamic range and emphasizes inter-cluster phenotypic homogeneity.

### 3.2. Clustering

A metric for similarity measures is required for clustering [28,32]. Root mean square (RMS) of Euclidean distance denoted as ‘*d*’ is used to measure the similarity between two records as,

(1) $$d\left(A,B\right)=\sqrt{\frac{\sum_{i=1}^{N}{\left(m_{A,i}-m_{B,i}\right)}^{2}}{N}}$$

where:

d(A,B): Distance between lactation dataset *A* and *B*
$m_{A,i}$: Amount of milk yield on $i^{th}$ day in lactation dataset *A*
$m_{B,i}$: Amount of milk yield on $i^{th}$ day in lactation dataset *B*
*N*: The total number of milking days

*K*-medoids clustering, a center-based clustering method, was used. It is advantageous for large datasets, although a pre-defined number of clusters is necessary [28,31,32]. Unlike the *k*-means algorithm, the *k*-medoids algorithm considers the median as the center of a cluster. Using a median instead of mean enhances robustness against outliers [40]. In the *k*-medoids algorithm, a cluster *S* is given as:

(2) $$S=\underset{S}{\arg\min}\sum_{A=1}^{k}\sum_{X\in S}d\left(X,\mu_{A}\right),$$

where:

$\mu_{A}$: A median dataset of a cluster *A*
$d(X,\mu_{A})$: Euclidean distance between lactation datasets X and $\mu_{A}$

The elbow method was adopted for the selection of the pre-defined number of clusters. The elbow method is a heuristic approach for determining the point where the local optimum is observed [41]. Six was the most appropriate cluster size with a low root mean square error (RMSE) in our experiments.

### 3.3. Characterization and Comparison of the Clusters

After clustering, differences in lactation and physiological characteristics (i.e., parity, peak milk yield, DIM at peak milk yield, total milk yield from 10 to 280 DIM, the day at 70 DIM in a lactation) were compared among the six clusters. The mean LCs of each cluster and the entire dataset were generated. Subsequently, the mean LCs were regressed to the three of the most popular LC models to investigate differences in estimated parameters and lactation characteristics (i.e., peak milk yield, DIM at peak milk yield, and persistency of milk production) among clusters. The models included the Wood model, an incomplete gamma function proposed by Wood [5], the Wilmink model, a model that combines exponential and linear decline function presented by Wilmink [6], and the Dijkstra model, a mechanical model presented by Dijkstra et al. [7], as shown in Table 3. The residuals of the models did not follow the normal distribution, rather shaped as bell curves.

An HPE Proliant Gen10 DL380 server equipped with 16 core CPUs (2.10 GHz) and 32 GB ECC DDR4 RAM was used for the computation. It took several minutes to group and train data. The least-square method was applied for the regression. Clustering algorithm was implemented in Python (v3.6). For the regression, the “SciPy” package (v1.3.0) was used.

### 3.4. Statistical Analysis

Analysis of variance was performed to evaluate the differences in milking characteristics among clusters. Differences in means were tested using Fisher’s Least Significant Difference. Statistical significance was set at p<0.05, and 0.05≤p<0.1 were considered trends.

Two fitting errors, $\epsilon_{f}$ and $\epsilon_{c}$ were measured to test fitting performance. $\epsilon_{f}$ is the difference between the regression curve and a representative LC of a cluster measured in RMSE. The mean value of the cluster is chosen as the representative LC. $\epsilon_{c}$ is the difference measured in RMSE between the regression curve and all the LCs in a cluster. When calculating $\epsilon_{c}$, all curves are each Z-normalized to compare shapes.

## 4. Results

The *k*-medoids clustering algorithm (k=6) grouped 330 datasets into six clusters of 119, 64, 50, 47, 38, and 12 datasets. The clusters were denoted (a)–(f) (Table 4). The largest cluster (a), had 36% of the datasets, while the smallest cluster (f) had 4% of the datasets, which represented the smallest dataset. Excluding the 70th DIM in a lactation, there were significant differences in parity, average daily milk yield, total milk yield from 10 to 280 DIM, peak DIM, and peak milk yield (p<0.01) among the clusters. Clusters (a) and (d), which had high proportions of multiparous cows, had a parity of 2.6, which was significantly higher than those of other clusters (p<0.05). In contrast, clusters (e) and (f) contained more primiparous cows than multiparous cows, and had lower average parities when compared with the other clusters (p<0.05). The daily average milk yield and total milk yield were the highest in cluster (a) (39.5 L and 10,713 L) and the lowest in cluster (e) (35 L and 9509 L) when compared to other clusters (p<0.05). Peak milk yield was the highest in cluster (d) (54 L), with an 11.6-L difference from cluster (e), which had the lowest peak yield (p<0.05). Clusters (a), (b), and (d) had peak yields at the early lactation period, while clusters (c), (e), and (f) had peak yields at the mid-lactation period. In cluster (e), which had the lowest peak yield, DIM at peak yield was 144 days, which was the latest among the six clusters (p<0.05).

**Figure 2:**
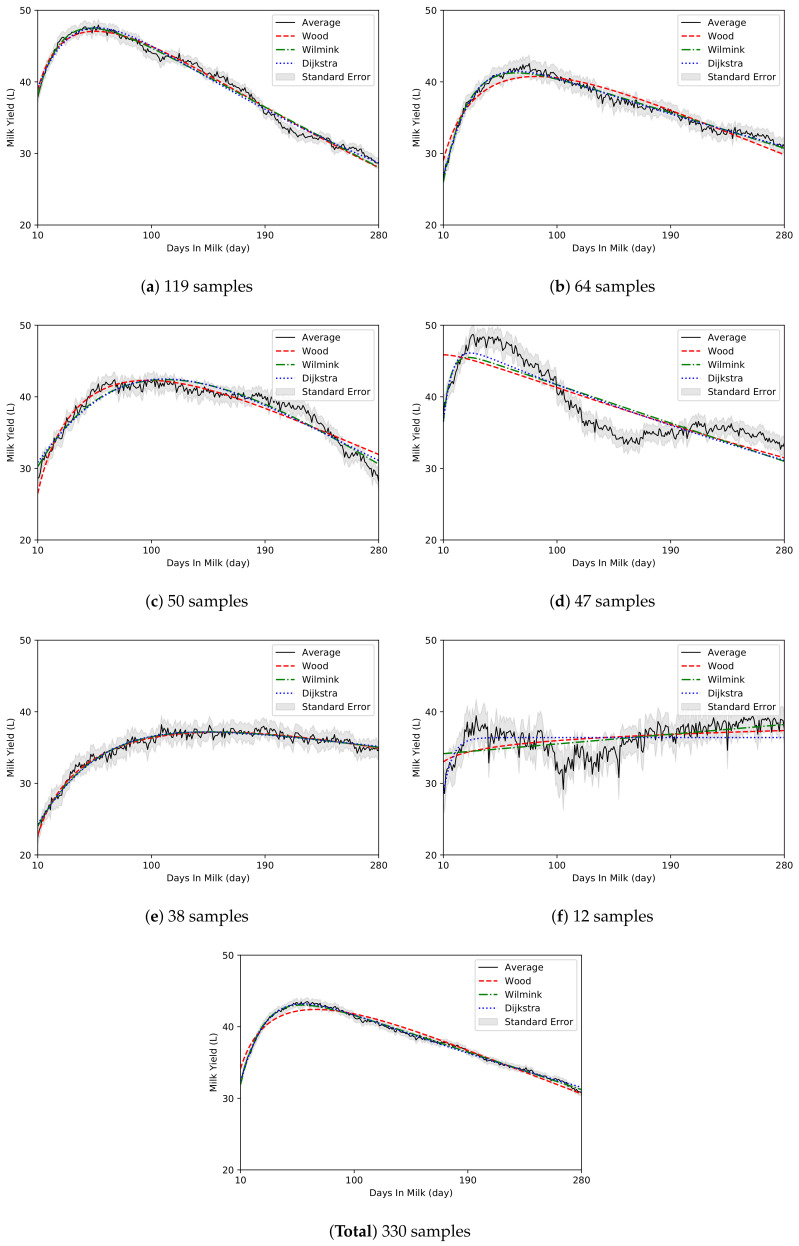
The averages of each cluster from 330 samples.

The regression graphs and model parameters of the three conventional models for the average lactation data in the clusters are presented in Figure 2 and Table 5, respectively. Clusters (a) and (b) curves displayed typical shape similar to that of the average LC of the 330 lactations, while clusters (d) and (f) curves had a different shape (Figure 2). Clusters (c) and (e) curves were gentle with lower peak milk yield than clusters (a) and (b). Cluster (d) curves exhibited rapid declines in milk production in the mid-lactation period after the high peak yields, and cluster (f) curves displayed an abnormal shape rather than a general LC. Cluster (f) curve did not fit properly in all three model. Distorted parameter estimates were also derived from the Wilmink model (Table 5). When the $\epsilon_{f}$s of the three models for the clusters were compared, the model with the lowest $\epsilon_{f}$ varied across the clusters (Wood model-cluster [e], Wilmink model-clusters [a] and [c], Dijkstra model-cluster [b]). The clusters (d) and (f), which exhibited abnormal shapes, had high $\epsilon_{f}$s (2.53 and 1.96, respectively) on average for all models, unlike other clusters. The $\epsilon_{c}$ for the lactation data of the samples within the cluster was the lowest in cluster (a) with the ideal curve shape, and the $\epsilon_{c}$ of cluster (f) was twice as high as that of cluster (a).

The estimated values of the parameters are listed in Table 6, except Wilmink model. Predicted values of peak milk yield were close between the Wood model and the Dijkstra model, but were underestimated by 6 L when compared to the actual lactation data. The greatest difference was 8 L, which was observed in cluster (d). Excluding in cluster (a), the calculated peak DIM value varied between the two models, and there was a gap of 15 days on average. The greatest difference between the models was 20 days, which was observed in cluster (d). Compared to the actual lactation value, the difference in the predicted peak DIM value from two models was relatively low, at 3 days in cluster (a) and (e), but high in cluster (d), at 34 days. Persistency, the relative declining rate at the half point between peak milk yield and the end of lactation, was the lowest in cluster (a) and the highest in cluster (e).

## 5. Discussion

Clustering of LC shapes yielded clusters (a), (b), (c), and (e), which accounted for 82% of the total individuals, and had typical LC shapes (Table 4). In particular, clusters (a) and (e) showed typical LC shapes of multiparous and primiparous cows, respectively. Primiparous cows generally have a flatter LC and have relatively high persistency, whereas multiparous cows exhibit rapid increases in daily milk yield from calving to the peak milk yield, followed by a significant decline [5,43]. Our study revealed significant differences in milking characteristics such as peak milk yield, peak time, and persistency (p<0.01). The results are consistent with the findings of previous studies [44,45,46,47,48,49], which reported that total milk yield in a 305-d lactation increases with an increase in the parity of Holstein cows. Particularly, some studies reported that peak milk yield increased dramatically from parity 1 to parity 2, and peak milk yield was observed at later periods in primiparous cows and earlier in multiparous cows [47,48]. Other studies have reported that milk yield is generally higher in multiparous cows than in primiparous cows, while persistency is often greater in the primiparous cows with less developed mammary glands [50,51,52]. In addition, most primiparous cows are not physically mature [53]. Since primiparous cow require nutrients for their own growth, their metabolic status is different from that of multiparous cows [54]. Such results support the observation that the clustering method used in the present study can discriminate milking characteristics such as total milk yield, peak milk yield, and DIM at peak yield, which vary depending on parity.

Clusters (d) and (f), which accounted for approximately 18% of the cows (Table 4), exhibited abnormal LC shapes (Figure 2). Cluster (f), which consisted of only 12 individuals (4%), had an LC shape with no peaks and no significant changes in milk production. The LC of cluster (d), which had high and rapid peaks, had an undulating shape due to a sharp decrease in milk yield during the mid-lactation period. Previous studies have reported that LCs with atypical shapes, such as no peak in LC, account for approximately 20∼30% individuals in datasets [2,9,13,14,15], which is consistent with the observations of the present study. Some studies have also reported very high peaks in the early lactation stages are accompanied by sharp decreases in milk yield during subsequent lactation periods, which could be due to metabolic stress and negative energy balance in some instances [55,56]. In addition, previous studies have reported that cows with high milk production and relatively early peak milk yields in early-lactation periods exhibit slow rates of recovery of body condition score, increased physiological stress, and have high risks of udder disease during mid- and late lactation periods [57,58,59]. The results suggest that the undulating shape of cluster (d) indicated energy unbalance or metabolic disorder. However, such atypical shapes (e.g., non-peak or undulating) of LCs could be caused by individual characteristics [2,9,60]. Arnal, et al. [61] classified LCs of dairy goats using principal component analysis and reported that LCs that were undulating in the mid lactation (120 DIM) period after peak yield were not associated with health problems or environmental factors. To facilitate precision dairy farming, it is critical to determine when such atypical LC shapes emerge, and whether they are attributable to variations, or health problems. Therefore, future studies should develop algorithms that can distinguish such variations or statuses.

The best fitting models are not the same in each clusters as shown in Table 5. The results are consistent with the those of previous studies [3,19]. Previous studies performed goodness of fit tests of the LC model for lactation data by grouping the data according to the period in which the milking data was obtained, the level of milk production, and parity, with the aim of finding a model that optimally describes lactation. Direct comparison on fitting accuracy of presented method to the previous studies is somewhat difficult to perform, since data collection duration, number of cows breed, metric of average milk yield and the data grouping method were not unified among the previous studies. The LC models in previous studies [3,19] were established from the selected groups considering ability of milk production and parity from a large breeding group. Abnormal populations were not considered for the LC model and excluded as exceptional cases. The previous approaches are not appropriate for studying the atypical cases of LC. The LC characteristics of atypical cases would be taken into account using the clustering method.

The three conventional LC models applied in the present study had high fitting errors for clusters with atypical shapes (Table 5). Cluster (d), which had the undulating shape, had the highest fitting error among the clusters, and cluster (f), which exhibited an abnormal shape without peaks, had distorted parameter estimates. Consequently, it was not possible to calculate peak yield, DIM, and persistence in cluster (f) using parameters derived from the model (Table 6). In addition, in cluster (d), the peak milk yield and DIM calculated using model parameters were significantly different from the actual milking data when compared with other clusters. Cluster (f) individuals accounted for only 4% of the individuals in the entire dataset and were less similar between individual LCs in the group. Therefore, the validation of individual data should be performed before further analyses (Table 5; $\epsilon_{c}$). However, cluster (d) individuals accounted for a considerable proportion of the total data (47 out of 330 cows). In addition, since the similarity between individual LCs in the group was comparable to the similarity observed within other clusters (Table 5; $\epsilon_{c}$), it can be considered as one of the LC types that can emerge in the commercial farms. In previous studies, LCs with atypical shapes have been omitted from initial datasets or their influence on the overall dataset has been diluted using mean values [16,17,18]. As mentioned above, such LC types are attributable to diverse factors ranging from simple individual differences to health problems [2,9,55,56,60], so more detailed investigations are required to determine their specific origins. However, in the present study, the conventional model had a high fitting error for LCs with atypical shapes, which is consistent with the findings of previous studies [9,15,62]. Therefore, it is necessary to develop a high-resolution model that can distinguish detailed features with high goodness of fit for diverse LC shapes.

In summary, clusters delineated from LC shape have different milking characteristics due to the varying proportions of multiparous and primiparous cows in each cluster. In addition, 18% of the individuals had atypical LC shapes. One of the clusters had an undulating LC shape and accounted for 14% of the individuals. This observation is presumably due to metabolic problems caused by rapid peaks and high production at the early stages of lactation. The fit of the model varied for each cluster following the fitting the three popular LC models to average lactation data clusters. We confirmed, however, that the conventional model is not suitable for clusters with atypical shapes due to high fitting errors. The method proposed in the present study may facilitate the identification and management of cows that require attention in herds. One of the practical applications of this study is to predict the daily milk production of individual cows using the cluster information. If the LC of currently milking cow follows a typically shaped cluster, then the regression models of the cluster can be thought as a predictor. The present study, however, was carried out using a limited amount of lactation data, so more commercial farm data should be collected to validate the various LC shapes. In addition, the correlations between LC shapes and the factors influencing such correlations should be investigated further based on biological and environmental data from individuals linked to lactation data. It is also necessary to investigate how many clusters of lactation data are appropriate for application in herd management. Finally, further studies should be conducted to develop a model that can mathematically explain LCs with various shapes.

## 6. Conclusions

This study described the development of a pattern-based clustering method for LCs using *k*-medoids algorithms. This methodology was able to successfully group the individual lactation data with similar milking characteristics (i.e., total milk yield, peak milk yield, and days in milk at peak yield). These results suggest that our methodology can facilitate the identification and management of cows that require attention in the herd.

## Figures and Tables

**Table 1:** Descriptive statistics of the raw data of milking records.

	Conventional		Automatic		Total	
	Milking System		Milking System			
	Mean	SE	Mean	SE	Mean	SE
Total milking days (day)	103,086		203,444		306,530	
Milking days (day/lactation)	207.00	5.99	243.94	5.20	230.13	3.98
Number of animals	287		437		724	
Number of total lactations	498		834		1332	
- Number of primiparous cows	191		269		460	
- Number of multiparous cows	307		565		872	
Parity	2.24	0.06	2.58	0.06	2.45	0.04
Daily milk yield (L/day/lactation)	30.75	0.52	32.82	0.39	32.06	0.31
Total milk yield (L/lactation)	7056	224	8349	189	7876	146

SE: standard error. SE calculated as the standard deviation divided by the square root of the number of lactations. The values of total milking days and the number of animals and lactations represent the total number, and the others describe the means and standard errors.

**Table 2:** Lactation statistics for the filtered data according to each parity.

Parity	Number of Lactations	Milking Days		Daily MY		Total MY	
	(Cows)	(Day)		(L/Day/Cow)		(L/Cow/Lactation)	
		Mean	SD	Mean	SD	Mean	SD
1	111	358.95	73.55	31.65	4.86	11,303	2649
2	101	377.30	78.56	36.00	5.41	13,545	3403
3	61	375.21	69.06	36.89	6.12	13,786	3310
≥4	57	406.58	81.59	37.09	6.60	14,981	3601

MY: milk yield; SD: standard deviation. All missing values are interpolated.

**Table 3:** Traditional regression models.

Model	Lactation Curve	
Wood (1967)	$y=a\cdot t^{b}\cdot e^{-c\cdot t}$	[5]
Wilmink (1987)	$y=a+b\cdot e^{-k\cdot t}+c\cdot t$	[6]
Dijkstra (1997)	$y=a\cdot e^{b\cdot \frac{1-e^{-c\cdot t}}{c}-d\cdot t}$	[7]

*y*: daily milk yield; *t*: time from parturition. All parameters *a*, *b*, *c* and *d* define the scale and shape of the LC. Wood model [5]: ’*a*’ represents the general production level, ’*b*’ represents the increasing period of the lactation before the peak yield, and ’*c*’ represents the decreasing period of the lactation after the peak yield. Wilmink [6] ’*a*’ represents the general production level, ’*b*’ represents the decreasing period of the lactation after the peak yield, and ’*c*’ represents the increasing period of the lactation before the peak yield, and ’*d*’ represents the peak day. Dijsktra [7] ’*a*’ represents the cell population at parturition (theoretical initial milk production), ’*b*’ represents the cell proliferation rate at birth, ’*c*’ represents the rate of decrease in cell proliferation during lactation, and ’*d*’ represents the cell death rate during lactation. All parameters are non-zero positive except *b* and *c* from Wilmink model. *b* and *c* in Wilmink model are non-zero negative.

**Table 4:** Descriptive statistics of each cluster and the entire dataset.

	Cluster						Total	*p*-Value
	(a)	(b)	(c)	(d)	(e)	(f)		
No. of lactations	119	64	50	47	38	12	330	
Primiparous	17	28	18	10	28	10	111	
Multiparous	102	36	32	37	10	2	219	
Parity	2.61 ^a^	2.16 ^c^	2.30 ^b^	2.57 ^a^	1.63 ^d^	1.25 ^e^	2.31	<0.001
	(0.12)	(0.17)	(0.19)	(0.20)	(0.20)	(0.18)	(0.07)	(0.16)
70 DIM	Aug. 1.	Sep. 29.	Oct. 10.	Aug. 20.	Sep. 30.	Jun. 30.	Aug. 31.	0.272
in a lactation (days)	(21.79)	(24.92)	(26.28)	(37.73)	(32.88)	(90.25)	(12.47)	(28.93)
MY (L/day)	39.53 ^a^	36.64 ^d^	38.20 ^c^	38.76 ^b^	35.09 ^e^	36.18 ^d^	38.03	0.006
	(0.61)	(0.96)	(0.93)	(1.02)	(1.13)	(1.69)	(0.39)	(0.88)
MY (L)	10,713 ^a^	9930 ^d^	10,351 ^c^	10,504 ^b^	9509 ^e^	9806 ^d^	10,305	0.006
	(164.73)	(261.38)	(252.30)	(274.96)	(305.46)	(459.28)	(104.65)	(238.49)
Peak DIM (days)	59.92 ^d^	86.25 ^c^	119.68 ^b^	54.94 ^e^	144.00 ^a^	119.92 ^b^	85.24	<0.001
	(2.73)	(4.61)	(8.42)	(6.11)	(9.67)	(25.73)	(3.01)	(6.01)
Peak MY (L)	53.08 ^b^	46.39 ^d^	47.14 ^c^	54.25 ^a^	42.70 ^e^	45.82 ^d^	49.59	<0.001
	(0.83)	(1.24)	(1.08)	(1.34)	(1.33)	(2.26)	(0.54)	(1.13)

MY: milk yield; DIM: days in milk. The number in parentheses represents a standard error, and particularly, the rightmost values mean the pooled standard error. Superscripts that are not common indicate significant differences within the cluster (p<0.05).

**Table 5:** The regression parameters and the RMSE results per each cluster.

Model	Parameter	Cluster						Total
		(a)	(b)	(c)	(d)	(e)	(f)	
Wood	a	24.6645	15.9437	12.5155	44.8198	12.2953	30.2838	22.1761
		(0.3161)	(0.3522)	(0.3401)	(2.1754)	(0.1697)	(1.3543)	(0.2843)
	b	0.2142	0.2738	0.3406	0.0170	0.2743	0.0374	0.2000
		(0.0037)	(0.0063)	(0.0076)	(0.0143)	(0.0038)	(0.0127)	(0.0037)
	c	0.0039	0.0033	0.0035	0.0016	0.0018	≈0	0.0029
		(≈0)	(0.0001)	(0.0001)	(0.0001)	(≈0)	(0.0001)	(≈0)
	$\epsilon_{f}$ (L)	0.6680	0.9529	1.1474	2.6746	0.5068 *	1.9778	0.6090
	$\epsilon_{c}$	0.6125	0.8121	0.8298	0.8827	0.8343 *	1.2126	0.9087
Wilmink	a	54.2924	46.0936	65.3834	47.6263	42.0509	6604.6668	47.5097
		(0.1707)	(0.1337)	(2.2023)	(0.3927)	(0.4718)	(0.1981)	(0.0581)
	b	−24.9865	−31.9822	−38.4509	−58.5113	−21.6106	−6570.6721	−25.8178
		(0.7756)	(0.6105)	(1.9260)	(35.6232)	(0.3697)	(0.1981)	(0.3245)
	c	−0.0937	−0.0549	−0.1200	−0.0594	−0.0247	≈0	−0.0582
		(0.0009)	(0.0007)	(0.0072)	(0.0023)	(0.0019)	(9.1156)	(0.0003)
	k	0.0494	0.0495	0.0124	0.1715	0.0195	≈0	0.0543
		(0.0020)	(0.0012)	(0.0009)	(0.0496)	(0.0008)	(0.0014)	(0.0008)
	$\epsilon_{f}$ (L)	0.6676 *	0.5240	1.0063 *	2.5458	0.5509	1.8807 *	0.2436 *
	$\epsilon_{c}$	0.6119 *	0.7866	0.8230 *	0.8682	0.8359	1.1974 *	0.9006 *
Dijkstra	a	34.0962	18.8021	28.2765	13.6532	21.3504	12.0427	25.1318
		(0.5020)	(0.3568)	(0.4192)	(8.0760)	(0.2697)	(7.7063)	(0.2315)
	b	0.0197	0.0467	0.0126	0.1950	0.0152	0.1686	0.0346
		(0.0011)	(0.0018)	(0.0005)	(0.1342)	(0.0007)	(0.1449)	(0.0009)
	c	0.0357	0.0506	0.0124	0.1525	0.0230	0.1525	0.0520
		(0.0015)	(0.0011)	(0.0010)	(0.0356)	(0.0009)	(0.0443)	(0.0008)
	d	0.0026	0.0015	0.0032	0.0016	0.0006	≈0	0.0016
		(≈0)	(≈0)	(0.0002)	(0.0001)	(≈0)	(0.0001)	(≈0)
	$\epsilon_{f}$ (L)	0.6933	0.4644 *	1.1214	2.3680 *	0.5784	2.0179	0.2479
	$\epsilon_{c}$	0.6131	0.7852 *	0.8316	0.8510 *	0.8371	1.2012	0.9010

RMSE: root means square error. Cluster means the RMSE calculation is based on each cluster, whereas Total is based on entire data. Models are fit using the averages from the records for each group. The RMSE is calculated between the average fitted model and each record in the group. The numbers in parentheses represent standard error. $\epsilon_{f}$ is calculated from based on the cluster average and the model. $\epsilon_{c}$ is the average RMSE between each Z-transformed sample in a cluster and a model; * The lowest error in each cluster.

**Table 6:** Peak yield, peak DIM, and persistency calculated using the obtained regression parameters.

	Cluster						Total
	(a)	(b)	(c)	(d)	(e)	(f)	
Wood model ^1^						
Peak yield (L)	46.96	40.65	42.34	45.87	37.10	N/A	42.34
Peak DIM (days)	54.92	82.97	97.31	10.63	152.39	N/A	68.97
Persistency	−0.0026	−0.0017	−0.0016	−0.0015	−0.0005	N/A	−0.0017
Dijkstra model ^2^						
Peak yield (L)	47.50	41.48	42.37	46.14	37.02	N/A	43.13
Peak DIM (days)	56.73	67.95	110.53	31.50	140.53	N/A	59.11
Persistency	−0.0025	−0.0015	−0.0020	−0.0016	−0.0005	N/A	−0.0016

Peak yield: peak milk yield; Peak DIM: days in milk at the peak milk yield; Persistency: relative rate of decline at the point halfway between peak milk yield and end of lactation [42]; N/A: not applicable; ^1^ The features of LC were calculated by the equations as follows [5,42]: Peak yield (L), $a*{\left(b/c\right)}^{b}*e^{-b}$; Peak DIM (days), b/c; Persistency, $b/t_{h}-c$; ^2^ The features of LC were calculated using the following equations [7,42]: Peak yield (L), $a*{\left(d/b\right)}^{d/c}*exp\left[\left(b-d\right)/c\right]$; Peak DIM (days), $c^{-1}ln\left(b/d\right)$; Persistency, $b*exp(-c*t_{h})-d$; * $t_{h}=\left(t_{m}+t_{f}\right)/2$; $t_{m}$, Peak DIM; $t_{f}$, length of lactation.
